# Molecular phylogeny and divergence times of Malagasy tenrecs: Influence of data partitioning and taxon sampling on dating analyses

**DOI:** 10.1186/1471-2148-8-102

**Published:** 2008-03-31

**Authors:** Céline Poux, Ole Madsen, Julian Glos, Wilfried W de Jong, Miguel Vences

**Affiliations:** 1 Department of Biomolecular Chemistry 271, Radboud University Nijmegen, PO Box 9101, 6500 HB Nijmegen, The Netherlands; 2 Division of Evolutionary Biology, Zoological Institute, Technical University of Braunschweig, Spielmannstr. 8, 38106 Braunschweig, Germany; 3 Vertebrate Department, Royal Belgian Institute of Natural Sciences, Vautierstraat 29, 1000 Brussels, Belgium; 4 Animal Breeding and Genomics Center, Wageningen University, PO Box 338, 6700 HB Wageningen, The Netherlands; 5 Animal Ecology and Conservation Biology Department, Biocenter Grindel and Zoological Museum, Martin-Luther-King Platz 3, 20146 Hamburg, Germany

## Abstract

**Background:**

Malagasy tenrecs belong to the Afrotherian clade of placental mammals and comprise three subfamilies divided in eight genera (Tenrecinae: *Tenrec*, *Echinops*, *Setifer *and *Hemicentetes*; Oryzorictinae: *Oryzorictes*, *Limnogale *and *Microgale*; Geogalinae:*Geogale*). The diversity of their morphology and incomplete taxon sampling made it difficult until now to resolve phylogenies based on either morphology or molecular data for this group. Therefore, in order to delineate the evolutionary history of this family, phylogenetic and dating analyses were performed on a four nuclear genes dataset (ADRA2B, AR, GHR and vWF) including all Malagasy tenrec genera. Moreover, the influence of both taxon sampling and data partitioning on the accuracy of the estimated ages were assessed.

**Results:**

Within Afrotheria the vast majority of the nodes received a high support, including the grouping of hyrax with sea cow and the monophyly of both Afroinsectivora (Macroscelidea + Afrosoricida) and Afroinsectiphillia (Tubulidentata + Afroinsectivora). Strongly supported relationships were also recovered among all tenrec genera, allowing us to firmly establish the grouping of *Geogale *with Oryzorictinae, and to confirm the previously hypothesized nesting of *Limnogale *within the genus *Microgale*. The timeline of Malagasy tenrec diversification does not reflect a fast adaptive radiation after the arrival on Madagascar, indicating that morphological specializations have appeared over the whole evolutionary history of the family, and not just in a short period after colonization. In our analysis, age estimates at the root of a clade became older with increased taxon sampling of that clade. Moreover an augmentation of data partitions resulted in older age estimates as well, whereas standard deviations increased when more extreme partition schemes were used.

**Conclusion:**

Our results provide as yet the best resolved gene tree comprising all Malagasy tenrec genera, and may lead to a revision of tenrec taxonomy. A timeframe of tenrec evolution built on the basis of this solid phylogenetic framework showed that morphological specializations of the tenrecs may have been affected by environmental changes caused by climatic and/or subsequent colonization events. Analyses including various taxon sampling and data partitions allow us to point out some possible pitfalls that may lead to biased results in molecular dating; however, further analyses are needed to corroborate these observations.

## Background

The Malagasy tenrecs belong to the Afrotheria, one of the four basal clades of placental mammals which have recently been recognized [1]. This ancient group of African origin is divided into two clades: the strongly supported Paenungulata, composed of the orders Sirenia (sea cows), Proboscidea (elephants) and Hyracoidea (hyraxes), and the Afroinsectiphillia [2], comprising the orders Afrosoricida (golden moles and tenrecs), Macroscelidea (elephant shrews) and Tubulidentata (aardvark) [3,4]. The tenrec family (Tenrecidae) comprises four subfamilies, the Potamogalinae from continental Africa, and the Tenrecinae, Geogalinae and Oryzorictinae from Madagascar. The Malagasy tenrecs are divided into eight genera and 30 species [5-8]. Based on morphology, tenrecs were previously grouped in the insectivorous order Lipotyphla, which has turned out to be biphyletic and now is split into the orders Eulipotyphla (hedgehogs, moles, shrews, solenodons) and Afrosoricida [9].

The Malagasy tenrecs have diversified into a spectacular radiation in terms of morphology, behavior, physiology and ecology. They show a high degree of adaptation to their niches (terrestrial, semi-arboreal, fossorial and semi-aquatic) and considerable convergence with other insectivores, notably shrews and hedgehogs. This made it difficult to understand the origin and phylogenetic relationships of this group on a morphological basis. The Tenrecinae (spiny tenrecs) include four genera (*Hemicentetes*, *Tenrec*, *Setifer*, *Echinops*), characterized by a spiny pelage and a large body size compared to the other tenrecs. Their monophyly is well established, even at the morphological level [10]. The branching of the four remaining genera (*Geogale*, *Oryzorictes*, *Limnogale *and *Microgale*), which share a shrew-like appearance and a small size, remains more open. Most earlier, molecular studies did not include more than five tenrec species [11-15], while Poux et al. [16] missed the large-eared tenrec (*Geogale*). Therefore, not all relations between and within the three subfamilies of Malagasy tenrecs have yet been firmly established. Only two recent studies, by Olson and Goodman [17] and Asher and Hofreiter [18], included all tenrec genera, but were unable to confidently resolve the position of *Geogale*, which suggests the necessity to expand the number of species and sequences for this family.

The island of Madagascar is a well-known biodiversity hotspot, displaying diverse and highly endemic amphibian, reptilian and mammalian faunas. The level of endemism reaches 95% for the non-flying vertebrates, and this level is mainly due to a few speciose endemic radiations [19-21]. Four clades of terrestrial endemic mammals are present, the lemuriform primates, the euplerine carnivores, the nesomyine rodents and the Malagasy tenrecs. Each of these clades represents one unique event of colonization from continental Africa, followed by several diversification events that gave rise to the actual Malagasy diversity [16,22]. The colonization of a new environment can be followed by an adaptive radiation, defined as a rapid succession of speciation events leading to a high ecological and phenotypic diversity within a lineage [23]. The study of adaptive radiations on islands or in lakes is essential for understanding processes of speciation and diversification [24-26]. Therefore, knowing the patterns and timing of the successive diversification events within endemic island clades, which, like tenrecs, display a broad ecological and morphological diversity, might help to better understand this phenomenon.

Apart from *Echinops telfairi*, for which the genome sequencing is in progress, there are only a limited number of sequences available in public databases to reconstruct a solid molecular phylogeny of the Malagasy tenrecs. In the present study we therefore selected exons from four independent nuclear genes that are widely used in mammalian phylogeny (ADRA2B, AR, GHR and vWF) in order to resolve tenrec phylogeny. This study is especially focused on understanding the phylogenetic position of the large-eared and the web-footed tenrecs, *Geogale *and *Limnogale*, respectively. In addition, we used a relaxed molecular clock timeframe to compare tenrec evolutionary patterns with defined adaptive radiation characteristics. Moreover, the influence of both taxon sampling and data partitioning on the accuracy of the estimated ages were assessed.

## Results and Discussion

### Afrotherian phylogeny

**Figure 1:**
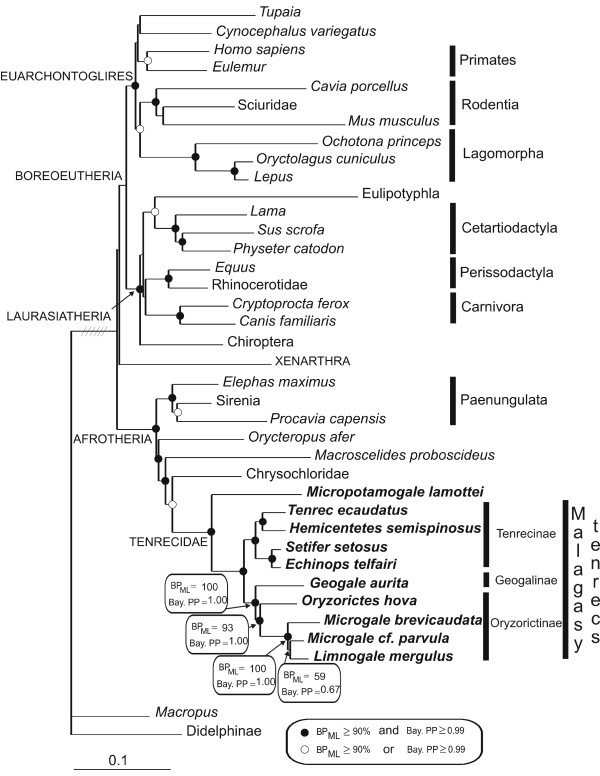
**Phylogenetic tree as inferred by maximum likelihood analysis of the concatenated 4,287-bp dataset**. Phylogenetic relationships of the investigated mammalian species were reconstructed using ADRA2B, AR, vWF and GHR sequences. Bayesian analyses result in an identical topology. Nodes receiving high support (BP ≥ 90% and PP ≥ 0.99) are marked with filled circles; open circles indicate that nodes received such high support with only one phylogenetic method (either BP or PP). Although the overall phylogenetic relationships as deduced from the present tree are consistent with the broadly accepted branching pattern of the mammalian tree [1], the phylogenetic position of the Eulipotyphla, displaying a high PP node support value, deviates from this consensus. The length of the branch connecting eutherians to the marsupial outgroup was reduced six times. Taxa not indicated by species name are represented by different species in the concatenated dataset, and the higher taxonomic unit is indicated (Table 1).

The overall phylogenetic relationships as deduced from the concatenated dataset are consistent with the now broadly accepted branching pattern of the mammalian tree [1] (Figure 1). The superordinal clades Euarchontoglires, Laurasiatheria and Afrotheria are highly supported, and within these clades most bootstrap percentages and posterior probabilities are also high. Afrotheria is now generally accepted as a natural group since molecular studies unanimously support its monophyly, using various methods [1,4,27-29]. In contrast, until now only few morphological synapomorphies, notably placental morphology [30], an increase in number of thoracolumbar vertebrae [31], and testicondy [32], appear to support this grouping. Afrotheria are divided into Paenungulata on one hand and the three remaining afrotherian orders (Afrosoricida, Macroscelidea and Tubulidentata) on the other hand. The most probable hypothesis concerning these remaining orders is their grouping within a clade called Afroinsectiphillia [1,3,28] within which the internal relationships remain unclear.

Within the paenungulate clade the Tethytheria (elephants + sea cows) are strongly supported by morphological and complete mitochondrial genome data [33,34]. Nuclear genes are ambiguous about this relationship and left the phylogenetic affinities between the three paenungulate orders essentially unresolved [1,14,35,36]. Our concatenated tree shows for the first time, based on nuclear genes, strong support for one of the three possible hypotheses: the grouping of Hyracoidea with Sirenia (PP = 0.99 and BP = 89). Bootstrap trees supporting alternative hypotheses exclusively group elephant with hyrax (BP = 11); Tethytheria is never recovered. All four genes independently support this result; the high support for the sea cow + hyrax grouping is therefore expectedly due to the synergy of these non-conflicting informations. To test whether our extensive taxon sampling within Tenrecidae may have improved the phylogenetic accuracy [37,38], all tenrecs but one (*Tenrec ecaudatus*) were removed from a new analysis. The results did not differ much; support for the Sirenia/Hyracoidea clade dropped negligibly in the concatenated analyses (PP = 0.98 and BP = 86). Interestingly, in a retroposon insertion analysis, Nishihara et al. [4] found one insertion supporting exclusively the grouping of hyrax with dugong. These authors dismissed the apparent synapomorphous hyrax-sea cow insertion as homoplastic, in favor of the morphological evidence for Tethytheria.

Similarly, the relations between the afroinsectiphillian orders have not yet been clarified, and conclusions vary in different studies. Mitochondrial data give highly inconsistent results [34,39], while mixed data tend to group golden moles and tenrecs with elephant shrews, together being the sister group of aardvark, with rather strong support [1,35,40]. Our data also support these results, as the Afrosoricida/Macroscelidea clade (= Afroinsectivora) is displayed with high confidence (PP = 1.00 and BP = 93), and Tubulidentata is found to be the sister group of this clade (PP = 1.00 and BP = 95). With a smaller dataset (only one tenrec) the support for the Afrosoricida/Macroscelidea clade slightly increased (PP = 1.00 and BP = 96). Hence, enlarged taxon sampling cannot explain our strong phylogenetic results within the afrotherian clade. All four genes separately displayed Afroinsectiphillia either as paraphyletic or weakly supported therefore the present results are not due to gene sampling biases. The retroposon analyses of Nishihara et al. [4] proposed the grouping of golden moles, tenrecs and aardvark, to the exclusion of elephant shrews, on the basis of two shared retrotransposons.

### Phylogenetic position of *Geogale aurita*

The large-eared tenrec (*G. aurita*) has been included until now in only two molecular studies, by Olson and Goodman [17] and by Asher and Hofreiter [18]. These two studies found two different results concerning its phylogenetic position. The first study, comprising three mitochondrial genes (ND2, 12s rRNA and tRNAvaline) and one nuclear marker (vWF exon 28), displayed, in a parsimony framework, the large-eared tenrec as the most basal of all Malagasy tenrecs. This result was not influenced by the inclusion of morphological characters in the analyses. Asher and Hofreiter [18], using exon 10 of the GHR gene and morphological data, found *Geogale *nested within the Oryzorictinae, as sister group of the *Microgale*/*Limnogale *clade.

**Figure 2:**
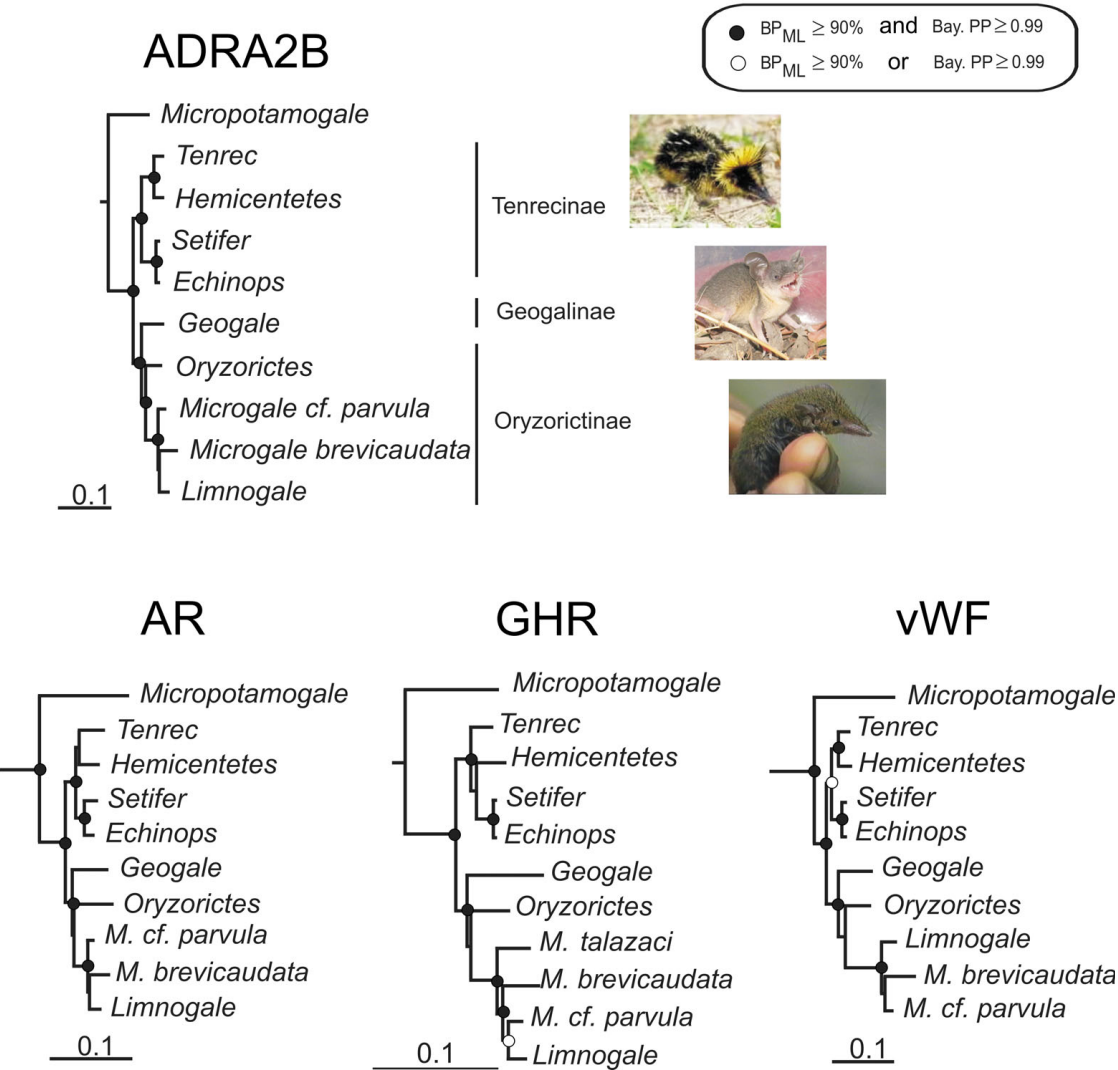
**Phylogenetic relationships of tenrecs as inferred by maximum likelihood analysis of the four separate datasets**. DNA matrix lengths were 1,101 bp for ADRA2B, 1,161 bp for AR, 852 bp for GHR and 1,173 bp for vWF. Bayesian analyses result in identical topologies. Nodes receiving high support (BP ≥ 90% and PP ≥ 0.99) are marked with filled circles; open circles indicate that nodes received a high support with only one phylogenetic method (either BP or PP). *M. talazaci *sequences were only available for GHR.

In the present study we also sequenced GHR exon 10 and vWF exon 28, and in addition the intronless gene for ADRA2B and the first exon of AR. For all genes separately the results were congruent in placing *Geogale *as sister group of the Oryzorictinae (Figure 2), although not always strongly supported: ADRA2B: PP = 1.00, BP = 96; AR: PP = 0.77, BP = 86; GHR: PP = 0.64, BP = 59; vWF: PP = 0.93, BP = 61. Concatenation of the four genes led to a stronger support for this node: PP = 1.00 and BP = 93 (Figure 1). The position of *G. aurita *as sister group of the Oryzorictinae (*Oryzorictes*, *Limnogale*, *Microgale*) seems thus strongly supported. However, the KH- and SH-tests (Table 1) did not completely confirm the strength of our results, showing that placing *Geogale *as the most basal Malagasy taxon (Olson and Goodman's hypothesis) was indeed significantly worse than our best tree, but placing it within the Oryzorictinae (Asher and Hofreiter's hypothesis) did not significantly change the likelihood of the topology.

**Table 1:** Results of the Shimodaira-Hasegawa test.

Trees	Phylogenetic hypothesis	-ln L	Δ-ln L	P
This study	*Geogale *sister group of Oryzorictinae	54619.52	best	
Asher and Hofreiter (2006)	*Geogale *nested within the Oryzorictinae	54632.08	12.56	P = 0.287
Olson and Goodman (2003)	*Geogale *sister group of all other Malagasy tenrecs	54677.72	58.20	P < 0.001

RELL and full option test give the same results. The Kishino-Hasegawa test applied to the following hypotheses leads to the same conclusions. Performing the tests including only the Afrotherian species in the analyses does not change the results either.

The differences with the results of Olson and Goodman [17] probably stem from the fact that we did not use the same phylogenetic methods and datasets, even though one of our markers was in common (vWF exon 28). However, their vWF (exon 28) sequences are not yet available in public sources like GenBank to be compared with ours. The different position of *Geogale *in the tree of Asher and Hofreiter [18] is more difficult to explain. Remarkably, their *Geogale *GHR sequence (Acc. Nr.: DQ202287) displays 18 differences with ours (10 synonymous and 8 non-synonymous substitutions). No mutations leading to unusual amino acid changes that might indicate sequencing errors could be detected. To try and explain the different *Geogale *GHR sequences we calculated Ka and Ks for each sequence pair of Malagasy tenrecs. The results showed that the sequence divergence between the two *Geogale *specimens was greater than between some of the other tenrec genera, like *Echinops*/*Setifer *and *Limnogale*/*Microgale *(Table 2). Moreover, the new *Geogale *sequence from this study was slightly more divergent in most comparisons than the one from the database (Table 2). This genetic diversity within *Geogale *could reflect that this genus might contain in fact more than one species. It may also be mentioned that the museum specimen used by Asher and Hofreiter [18] was collected at the southwest coast of the island (Lamboharana, voucher number MCZ 45044), whereas our specimen (voucher number MVZ mammal # 220648) was sampled in the central west in the Menabe area. Considering photos of living *Geogale *available to us from the south-west (by W. R. Branch) and the central-west (by R. Nincheri and ourselves), the central western specimens appear to have a less golden-colored fur and in general a more gracile habitus, but it is unclear whether this may reflect a difference between coloration of adults versus subadults. Clearly, a detailed taxonomic study is needed to confirm whether these differences are constant and the populations may represent two distinct species. Furthermore, a single record of *Geogale *exists also from the east coast near Fenoarivo. This specimen has been described as subspecies *Geogale aurita orientalis *by Grandidier and Petit [41], but the status of this taxon has remained obscure. It may be a candidate nomen to be elevated to species rank if *Geogale aurita *is demonstrated to consist of more than one species.

**Table 2:** Ks and Ka calculated for each pair of Malagasy tenrec GHR sequences.

	*Tenrec*	*Setifer*	*Hemicent*.	*Echinops*	*Oryzorictes*	*Geogale *A	*Geogale *B	*M.talazaci*	*M.brevi*.	*M. cf. parvula*	*Limnogale*
*Tenrec*		**0.02**	0.03	**0.02**	0.04	0.06	0.06	0.06	0.07	0.06	0.06
*Setifer*	0.09		0.03	**0.00**	0.03	0.07	0.07	0.06	0.07	0.07	0.06
*Hemicent*.	0.08	0.11		0.03	0.04	0.07	0.07	0.07	0.07	0.06	0.07
*Echinops*	0.09	**0.02**	0.11		0.03	0.06	0.06	0.06	0.07	0.06	0.06
*Oryzorictes*	0.13	0.19	0.17	0.19		0.05	0.06	0.05	0.06	0.05	0.05
*Geogale *A	0.14	0.19	0.16	0.19	0.15		**0.02**	0.07	0.07	0.06	0.06
*Geogale *B	0.16	0.21	0.19	0.21	0.17	**0.05**		0.07	0.08	0.07	0.07
*M.talazaci*	0.15	0.22	0.21	0.22	0.20	0.19	0.20		0.04	0.03	0.03
*M.brevi*.	0.17	0.21	0.19	0.22	0.21	0.21	0.24	0.15		0.03	0.04
*M.cf.parvula*	0.14	0.17	0.14	0.17	0.17	0.17	0.20	0.09	0.09		**0.02**
*Limnogale*	0.14	0.17	0.14	0.17	0.18	0.20	0.22	0.12	0.10	**0.03**	

Ks (i.e., number of synonymous substitutions per synonymous site) are given in the lower left part of the table and Ka (i.e., number of nonsynonymous substitutions per nonsynonymous site) in the upper right part. The divergence between the two *Geogale *GHR sequences (underligned) is greater than or equal to that between some other tenrec species (bold). *Geogale *A is the sequence from the database (Acc. Nr.: DQ202287), *Geogale *B is our sequence. *Hemicent*. stands for *Hemicentetes*, and *M. brevi*. for *Microgale brevicaudata*.

Further phylogenetic analyses of the GHR dataset, including both *Geogale *sequences or removing all segregating sites between the two sequences, led to the same result as obtained by Asher and Hofreiter [18], i.e. *Geogale *nested within the Oryzorictinae. The phylogenetic position of *Geogale *as sister group of Oryzorictinae was only obtained when our sequence alone was used. However, both *Geogale *sequences always grouped together, confirming the identity of our sequence. These results, in combination with the fact that the Oryzorictinae/Geogalinae clade radiated very fast, might make it difficult to reach a final consensus on the evolution of *Geogale*.

From a morphological point of view the phylogenetic relation between *Geogale *and the Oryzorictinae has never been clear. Although most studies gave unresolved results [[10], Olson [1999] in [17,18]], two were concordant with ours [42,43], while none has ever argued that *Geogale *was either the sister group of all Malagasy tenrecs or the sister group of the *Limnogale*/*Microgale *clade. Salton and Szalay [43] reached the conclusion that the tarsal morphology of *Geogale *warrants its status as a separate subfamily, and suggested its closer affiliation with Oryzorictinae than with Tenrecinae.

Three genera of fossil tenrecids – *Erythrozootes*, *Protenrec *and *Parageogale *– from the Kenyan and Namibian Miocene (16–24 Mya; Million years ago) have been discovered until now [44-46]. As *Parageogale *is thought to be the sister group of the extant *Geogale aurita *[45], these data would suggest a more complex dispersal history than the "one time dispersal event" deduced from the monophyly of Malagasy tenrecs. Asher and Hofreiter [18] were the first to include these three fossil tenrecids in a phylogenetic framework. Their result confirmed the position of the Kenyan fossils as *Geogale'*s closest relatives. However, alternative hypothesis (e.g., monophyly of the Malagasy tenrecs) could not be ruled out indicating the uncertainty of the *Parageogale*/*Geogale *affinity. Recent studies have argued that the sweepstakes dispersal model (dispersal with small and random probability of success) from Africa to Madagascar suffers from many inconveniences, among which the fact that prevailing winds and currents between Africa and Madagascar would be much more likely to favor transports from the island to the African continent, rather than the reverse route [47,48]. Therefore, if a second dispersal event ever occurred it was most probably from Madagascar to Africa. Olson and Goodman [17] suggested a basal position of *Geogale *among Malagasy tenrecs and argued that, if true, this would only imply a minimum of two dispersal events, whereas any other scenario would require at least three. However, a back dispersal of *Parageogale *from Madagascar to Africa would only assume a second dispersal event, independent of the phylogenetic position of *Geogale*.

### Phylogenetic position of *Limnogale mergulus*

Due to its semi-aquatic life style, shared with the African Potamogalinae, the determination of the phylogenetic relationship of *Limnogale*, the web-footed tenrec, has led to controversies. Its specialized morphological features brought some authors to the conclusion that *Limnogale *was either sister group of the Potamogalinae [10] or sister group of all other Malagasy tenrecs [42], the semi-aquatic behavior then being seen as an ancestral state and a key element to facilitate over-water dispersal. In contrast, other morphological studies challenged this view by affirming that *Limnogale *had closer relationships to the shrew tenrecs (*Microgale*), and that the semi-aquatic behavior was an example of convergence acquired twice during tenrec evolution [Guth et al.[1959] in [17], Olson [1999] in [17]]. This strong affinity between *Limnogale *and *Microgale *has recently also been supported by a study of hind limb muscles [49]. These authors argue that *Limnogale *may have been derived from a *Microgale*-like terrestrial ancestor. Molecular studies have now confirmed this last hypothesis [16-18]. Supporting the hypothesis of Olson and Goodman [17], our study shows that the semi-aquatic *Limnogale *is actually nested within the shrew tenrec genus and not a sister clade of it (Figure 1), now with more elaborate analyses and strong support from four nuclear genes.

The phylogenetic supports displayed in the present study are quite low, even with the concatenated dataset (PP = 0.67 and BP = 59), probably due to the fact that the *Microgale/Limnogale *clade may have radiated very fast (Figure 1). Only one gene, GHR, presents a high PP of 0.99 for the cluster of *Microgale cf. parvula*/*Limnogale mergulus *(Figure 2). The sequencing of more shrew tenrec species (a total of 21 species has been recorded [5-8]) might help to resolve this issue, and subsequently to understand the morphological evolution of the aquatic specialization of the web-footed tenrec.

### Tenrec diversification timing

**Figure 3:**
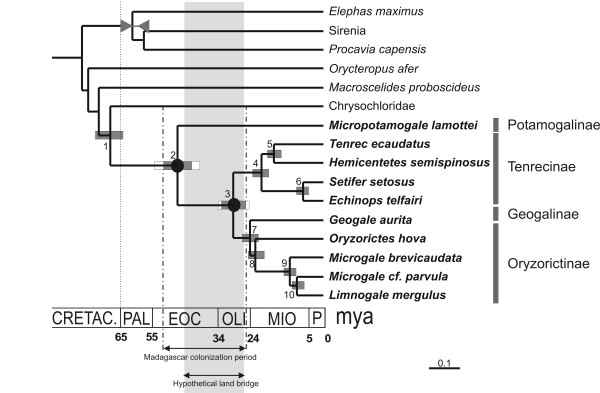
**Timing of tenrec speciation events and Madagascar colonization**. Tree topology as in Figure 1. Divergence times were estimated from the concatenated dataset by a Bayesian relaxed molecular clock method, with six time constraints from fossil calibrations (see Material and Methods). One of them, the paenungulate radiation is represented on the chronogram. Black circles indicate the divergence from the non-Malagasy sister group (node 2) and the initial divergence of Malagasy tenrecs (node 3). Standard deviations are indicated by grey bars, and 95% credibility intervals by open bars. The period of a putative land bridge between Madagascar and Africa at 45–26 Mya [53] is shaded.

Only three studies have previously assessed the timing of tenrec diversification, mainly to understand their colonization pattern [13,14,16]; none comprised a taxon sampling broad enough to delineate the successive tenrec speciation events. The study by Douady et al. [13] was based on a linearized tree method and suggests an early diversification of Tenrecs as compared to the other studies (for the present study see Figure 3), which are based on Bayesian methods and partially overlapping gene sampling (Table 3). Consequently, the results of the latter three studies are, as can be expected, rather similar. The present study, with the broadest taxon and gene sampling, estimates the tenrecs/golden mole split at 69 ± 4 Mya, followed by the divergence between African and Malagasy tenrecs at 47 ± 4 Mya. The Malagasy tenrec radiation began 29 ± 3 Mya, and several diversification events spread over time gave rise to the totality of Malagasy tenrec genera around between 20 ± 1 Mya and 7 ± 1 Mya (Table 3 and Figure 3). These datings are slightly older than previously calculated. The only gene difference between this study and Poux et al. [16] is the inclusion of the GHR gene. Removing it from the calculations led to dates even a little older and with wider confidence intervals (Table 3).

**Table 3:** Comparison of estimated Malagasy tenrec divergence times (in Mya).

Clade and node number	[13] ^a^	[14] ^b^		[16] ^c^		This study 9 partitions		This study without GHR	
	
	Age	Age ± SD	95% CI	Age ± SD	95% CI	Age ± SD	95% CI	Age ± SD	95% CI
Tenrecidae/Chrysochloridae, 1	-	63 ± 5	53–72	67 ± 5	58–76	69 ± 4	61–77	71 ± 4	62–80
Malagasy tenrecs/Potamogalinae, 2	51–55	43 ± 5	34–52	42 ± 4	34–50	47 ± 4	40–55	45 ± 4	37–54
Malagasy tenrec radiation, 3	37	-	-	25 ± 3	20–32	29 ± 3	24–35	30 ± 3	24–37
Tenrecinae radiation, 4	18–44	16 ± 3	11–22	18 ± 2	13–23	20 ± 2	16–25	21 ± 3	16–26
*Tenrec*/*Hemicentetes *split, 5	-	-	-	13 ± 2	10–18	16 ± 2	12–21	15 ± 2	11–20
*Setifer*/*Echinops *split, 6	*-*	-	-	6 ± 1	4–9	7 ± 1	4–9	8 ± 2	5–11
Geogalinae/Oryzorictinae split, 7	-	-	-	-	-	24 ± 3	19–29	24 ± 3	19–31
Oryzorictinae radiation, 8	-	-	-	19 ± 3	14–25	22 ± 3	17–27	22 ± 3	17–28
*Microgale *radiation, 9	*-*	-	-	-	-	11 ± 2	8–15	11 ± 2	7–15
*Microgale*/*Limnogale *split, 10	*-*	-	-	-	-	9 ± 1	6–12	9 ± 2	6–13

Node numberd as in Figure 3. SD: standard deviations; CI: credibility intervals; – Nodes not present in the study.

^a^Age estimated from vWF, 12s and 16s

^b^Age estimated from vWF, ADRA2B, BRCA1

^c^Age estimated from vWF, ADRA2B, AR

Because the GHR influence on the dating was very small, the difference in taxon sampling between the two studies might be responsible for the different outcomes [50]. In the present study carnivores and primates were less extensively sampled, whereas Afrosoricida were better represented than in Poux et al. [16]. We therefore compared for these three clades the age inferences in Poux et al. [16] and in the present study, with or without GHR (Table 4). The conclusion is that the age of a given node tends to become older when the taxon sampling around this node (or descending from it) increases. This phenomenon has already been described by Yoder and Yang [51] when assessing the timing of evolution of mouse lemurs. They suspected that these incongruences were due to the model used [52], which breaks down the path from a tip of the tree to the root (or ancestral node) into identically distributed segments. Such a prior would tend to push divergence time within the clade under study towards unrealistically old ages. Comparing the priors of divergence times between both large and small datasets, they reached the conclusion that the too old priors of the larger dataset had influenced the posterior estimates, which became older as well. This also is the pattern we can see comparing the priors of Poux et al. [16] with the ones of the present study (dataset without GHR). In both studies the time estimate differences were not dramatic, but they could have a problematic effect for studies requiring more precise estimates.

**Table 4:** Posterior estimates of divergence times (Mya ± standard deviation) inferred from the concatenated datasets.

Radiation	Calibration time frame (Mya) ^a^	[16]^b^	This study without GHR^b^	This study^b^
Primates	none	**79.1 ± 4.7**	73.5 ± 4.8	75.5 ± 4.3
Carnivora	50–63	**55.6 ± 3.1**	54.7 ± 3.0	53.3 ± 2.4
Afroinsectiphillia	none	73.7 ± 4.0	**77.3 ± 3.9**	**76.5 ± 3.6**

Bayesian relaxed molecular clock method was used. Ages in bold indicate the study in which the corresponding order was more extensively sampled. The result shows that increasing the sampling size pushes the ages towards older estimates. In this analysis rodents could not be taken into account because of sampling incongruences between the two studies.

^a ^Paleontological time constraints used as calibrations.

^b ^The results of MODELTEST were used to define the partitioning; the three studies are therefore directly comparable.

**Figure 4:**
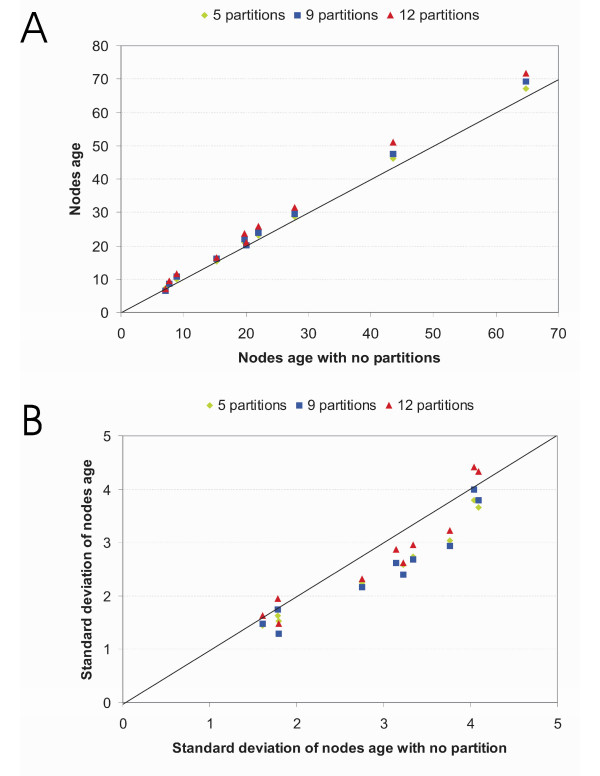
**Congruence of divergence time estimates (A) and their associated standard deviations (SD) (B) when calculated with different partition types**. The X-axis represents the estimates without partitioning and the Y-axis the ones with 5, 9 or 12 partitions (see Methods). The age estimates increase with the number of partitions (A) and the SDs are larger for extreme numbers of partitions (none and 12 partitions) (B). For clarity purpose only the age estimates relative to tenrecs are displayed in these graphs; however the estimated ages and SDs in the rest of the tree give the same results.

The influence of data partitioning was tested as well. The ages of the nodes in the phylogenetic tree increased with the number of partitions (Figure 4A), and the smallest standard deviations (and therefore confidence intervals) were reached for the less extreme numbers of partitions (Figure 4B). However, for the present study, differences in taxon sampling or partitioning did not affect our conclusions, as the various analyses displayed fairly similar results, showing reciprocal overlaps. This is to our knowledge the first time that the influence of data partitioning on dating results has been empirically pointed out. More investigations are needed to generalize and clearly understand the underlying causes of this result. One might however suppose that the differences between the various partitions could increase with the number of genes included in an analysis. Consequently, these results show that it is important, in order to calculate datings as accurately as possible, to select the right manner of partitioning the data: too few or too many partitions might lead to biased results.

To exclude the possibility that individual calibration constraints may bias our dating analyses, we repeated them after removing each calibration point in turn following [16]. Hereby we could check whether the excluded calibration constraint was accurately estimated by the remaining ones. All datings remained highly congruent when any of the six calibration points was removed. The average percentage difference between the main analysis and the ones with only 5 constrained nodes ranges between 0.1 and 0.8 percent. Only the paenungulate calibration seems to have a somewhat larger impact on the dating as its removal from the analysis increases the estimated node age by 4.8 percent. This influence is however too slight to have an impact on our conclusions (Additional files 1 and 2). Moreover, the calibrations were reciprocally compatible: the remaining five calibrations always recovered a posterior estimate (± SD) for the excluded node within the time window independently obtained from the corresponding fossil evidence (Additional files 1 and 2).

Since *Geogale *has been hypothesized by Olson and Goodman [17] to be the first Malagasy tenrec genus to have diverged, its absence from Poux et al. [16] was a problem for drawing final conclusions about tenrec colonization timing. It now appears that *Geogale *is nested within the Malagasy tenrec clade, and therefore plays no role when estimating the period of colonization. Consequently, the window of colonization of Madagascar by tenrecs could not be narrowed. As previously concluded in Poux et al. [16], the tenrec colonization time completely overlaps with the hypothetical time of existence of a land bridge crossing the Mozambican channel (26–45 Mya; [53]) (Figure 3), which however is highly controversial [54].

Adaptive radiation often occurs when a species is introduced into a new environment, such as an island. One might therefore expect that the majority of the diversification events within the Malagasy tenrecs would have occurred soon after colonization. However, no such pattern of a diversification burst can be seen at the root of the Malagasy tenrecs, and speciation events seem to be spread through time (Figure 3). This could result from two possible scenarios: either Malagasy tenrecs may actually have experienced a fast adaptive radiation, but most of the resulting taxa are now extinct, or all genera appeared indeed at different periods as a result of a slower speciation rate than expected in case of adaptive radiations. Morphologically, one might speak about adaptive radiation of Malagasy tenrecs, but these morphological adaptations do not seem to have developed within a short time span just after the colonization of Madagascar. The most striking example is the semi-aquatic specialization of the genus *Limnogale*, which dates at most from 11 Mya, i.e., 20–38 My after the colonization of the island. The genus *Microgale *is by far the most speciose amongst tenrecs, being represented by 21 species [5-8], while the remaining genera may not comprise more than one species. The acceleration of the molecular evolutionary rates on the internal branches leading to and within this genus (calculated with MULTIDIVTIME on the tree presented in Figure 1), associated with both a poor phylogenetic resolution between the few *Microgale *species (Figs 1 and 2) and its recency among the tenrec genera (Figure 3 and Table 5), suggests that there has been a fast radiation around 11 Mya that gave rise to the current diversity of *Microgale*. It is interesting to note that the other two endemic mammalian Malagasy genera for which radiation times have been assessed apparently diverged around the same time as *Microgale*: *Eulemur *at 9.7 Mya and *Microcebus *at 8.7–12 Mya [51]. However, not enough data are yet available to confirm this parallel radiation phenomenon.

**Table 5:** Taxonomic sampling and accession numbers of the four nuclear genes.

	Species	ADRA2B	AR	GHR	vWF
EUTHERIA					
RODENTIA					
Muridae	*Mus musculus*	L00979	NM_013476	M33324	AJ238390
Caviidae	*Cavia porcellus*	AJ271336	AJ893531	AF238492	AJ224663
Sciuridae	*Marmota/Sciurus *^1^	AJ315942	AM905334*	AF332032	J224671
LAGOMORPHA					
Leporidae	*Oryctolagus cuniculus*	Y15946	AJ893533	AF015252	U31618
	*Lepus *sp. ^2^	AJ427254	AJ893534	AF332016	AJ224669
Ochotonidae	*Ochotona princeps*	AJ427253	AJ893535	AF332015	AJ224672
PRIMATES					
Lemuridae	*Eulemur *sp.^3^	AJ891059	AJ893537	AF540627	AJ891087
Hominidae	*Homo sapiens*	M34041	M27423	X06562	X06828
SCANDENTIA	*Tupaia *sp. ^4^	AJ251187	AM905335*	AF540643	U31624
DERMOPTERA	*Cynocephalus variegatus*	AJ251182	AM905340*	AF540625	U31606
CARNIVORA					
Canidae	*Canis familiaris*	AJ891051	AF197950	AF133835	L16903
Felidae	*Cryptoprocta ferox*	AJ891056	AJ893549	AY928733	AJ891085
PERISSODACTYLA					
Rhinocerotidae	*Ceratotherium/Diceros *^5^	AJ251184	AJ893553	AM905343*	U31604
Equidae	*Equus *sp. ^6^	Y15945	AJ893554	AF392878	U31610
CETARTIODACTYLA					
Camelidae	*Lama *sp. ^7^	AJ315941	AJ893555	AM905349*	AF108835
Suidae	*Sus scrofa*	AJ251177	AF161717	X54429	S78431
Physeteridae	*Physeter catodon*	AJ427417	AJ893556	AM905344*	AF108834
CHIROPTERA	*Cynopterus/Pteropus *^8^	AJ251181	AM905339*	AF392893	U31605
EULIPOTYPHLA	*Erinaceus/Crocidura *^9^	Y12521	AJ893557	AF392882	AY057834
XENARTHRA	*Myrmecophaga/Cyclopes *^10^	MTR427373	AJ893558	AF392875	MTR278157
SIRENIA	*Trichechus/Dugong *^11^	AJ251109	AJ893559	AF392891	U31608
PROBOSCIDEA	*Elephas maximus*	Y12525	AJ893560	AF332013	U31611
HYRACOIDEA	*Procavia capensis*	Y12523	AJ893561	AF392896	U31619
TUBULIDENTATA	*Orycteropus afer*	Y12522	AJ893563	AF392892	U31617
MACROSCELIDEA	*Macroscelides proboscideus*	Y12524	AM905337*	AF332014	AY310893
AFROSORICIDA					
Chrysochloridae	Amblysomus/Chrysospalax 12	Y12526	AJ893562	AF392877	U97534
Tenrecidae					
Tenrecinae	Setifer setosus	AJ891077	AJ893566	DQ202292	AJ891098
	*Echinops telfairi*	Y17692	AJ893565	AF392889	AF076478
	*Tenrec ecaudatus*	AJ251108	AJ893564	AF392890	AF390536
	*Hemicentetes semispinosus*	AJ891065	AJ893567	DQ202288	AJ891093
Oryzoryctinae	*Oryzorictes hova*	AJ891074	AJ893568	AF392886	AJ891097
	*Microgale talazaci*	-	-	AF392885	-
	*Microgale brevicaudata*	AJ891072	AJ893569	AM905345*	AM905350*
	*Microgale *cf. *parvula*	AM905341*	AM905336*	AM905346*	AM905351*
	*Limnogale mergulus*	AJ891069	AJ893570	DQ202289	AJ891096
Geogalinae	*Geogale aurita*	AM905342*	AM905338*	AM905347*	AM905352*
Potamogalinae	*Micropotamogale lamottei*	AJ251107	AJ893571	DQ202290	AF390538
MARSUPIALIA					
DIDELPHIMORPHIA	*Didelphis/Monodelphis *^13^	Y15943	AJ893572	AF238491	AF226848
DIPROTODONTIA	*Macropus *sp. ^14^	AJ251183	AJ893573	AM905348*	AJ224670

^1 ^*Sciurus vulgaris *(ADRA2B, AR) combined with *S. niger *(GHR) and *Marmota monax *(vWF)

^2 ^*Lepus crawshayi *(ADRA2B, AR, vWF) combined with *L. capensis *(GHR)

^3 ^*Eulemur fulvus fulvus *(ADRA2B, AR, vWF) combined with *E. coronatus *(GHR)

^4 ^*Tupaia tana *(ADRA2B, AR, GHR) combined with *T. glis *(VWF)

^5 ^*Diceros bicornis *(ADRA2B, AR, GHR) combined with *Ceratotherium simum *(vWF);

^6 ^*Equus caballus *(ADRA2B, AR, GHR) combined with *E. asinus *(vWF)

^7 ^*Lama pacos *(ADRA2B, AR, GHR) combined with *L. glama *(vWF)

^8^*Cynopterus sphinx *(ADRA2B, AR, vWF) combined with *Pteropus vampyrus *(GHR)

^9 ^*Erinaceus europaeus *(ADRA2B, AR, GHR) combined with *Crocidura russula *(vWF)

^10 ^*Myrmecophaga tridactyla *(ADRA2B, vWF, GHR) combined with *Cyclopes didactylus *(AR)

^11 ^*Trichechus manatus *(ADRA2B, AR, GHR) combined with *Dugong dugon *(vWF)

^12 ^*Amblysomus hottentotus *(ADRA2B, AR, vWF) combined with *Chrysospalax trevelyani *(GHR)

^13 ^*Didelphis marsupialis *(ADRA2B, AR) combined with *D. virginiana *(vWF) and *Monodelphis domestica *(GHR)

^14 ^*Macropus rufus *(ADRA2B, AR, GHR) combined with *M. giganteus *(vWF)

Upperscore numbers (^1–14^) refer to taxa for which sequences from different species were combined in the concatenated analysis. * New sequences from the present study. The full alignment is available from Treebase (accession number M3679).

Even though the colonization of Madagascar by tenrecs might have taken place during the Eocene, the radiation of the extant species started after Madagascar reached its current geographical subtropical location during the early Oligocene [55], with warmer climatological conditions probably similar to the actual ones [56]. The colonization of Madagascar by carnivores and rodents took place at the end or just after the Oligocene, around 20–23.5 Mya for rodents, and 19–26 Mya for carnivores (data taken from [16] in order to compare results inferred from similar datasets and methods). These dates are quite close to the periods of appearance of extant tenrec genera: the radiation of Tenrecinae and the split between *Tenrec *and *Hemicentetes *occurred 20 ± 2 Mya and 16 ± 2 Mya, respectively; *Geogale *split from the Oryzorictinae 24 ± 3 Mya; and *Oryzorictes *separated from *Microgale *22 ± 3 Mya. So five out of the seven tenrec genera (*Limnogale *is taken here as a *Microgale*) diverged soon after the colonization of Madagascar by carnivores and rodents. These new colonizations may have altered the ecological conditions, and thereby induced speciation within tenrecs, either by predation pressure (carnivores) or by interspecific niche competition (rodents).

## Conclusion

The complete phylogeny of the Malagasy tenrec genera has now been resolved with strong support. These results should lead to a revision of the taxonomy with regard to the genus *Geogale *(if it comprises more than one species) and the *Limnogale*/*Microgale *clade (if this last genus is truly paraphyletic). This solid phylogenetic and dating framework shows that the major morphological specializations of the tenrecs are not the result of fast adaptive radiations just after colonization, but would as well have been affected by ecological changes caused by climatic and/or subsequent colonization events; however, more work is still needed to understand the role of possible biotic interactions on the speciation processes of Malagasy tenrecs.

## Methods

### Sampling, DNA amplification and sequencing

Fragments of the intronless gene of the alpha 2B adrenergic receptor (ADRA2B), of exon 1 of the androgen receptor (AR) gene, of exon 10 of the growth hormone receptor (GHR) gene, and of exon 28 of the von Willebrand factor (vWF) gene were amplified and sequenced. These genes were selected because (i) they are located in the nuclear genome, as single-copy genes (in at least human and mouse), (ii) a considerable number of sequences are already available for all four genes and have been useful in mammalian phylogeny, and (iii) they are functionally and genetically unrelated. We selected for each of the four genes 38 mammalian species to represent (i) all genera of Malagasy tenrecs, and at least two species of the very diverse genus *Microgale*, in order to assess the phylogenetic position of *Limnogale*, (ii) the continental African sister group (Potamogalinae) of the Malagasy tenrecs, (iii) groups needed for multiple calibrations of the molecular clock, (iv) at least one species from each eutherian order (but for Pholidota), and (v) appropriate marsupial outgroups. A total of 19 new sequences were obtained, and complemented with 134 sequences from GenBank (Table 5).

Genomic DNA was isolated from ethanol-preserved tissue, following the protocols of the Wizard^® ^SV Genomic DNA Purification System (Promega). Fragments of the ADRA2B and AR genes were amplified using previously published primers [16,57]. New primers were designed for vWF and GHR (see Additional file 3). For these last genes PCR reactions were performed on 50–200 ng DNA with Expand DNA polymerase (Expand High Fidelity PCR system, Roche) using the following program: 2 min at 94°C; 30–35 cycles of 15 sec at 94°C, 1 min at 60°C and 1 min 30 sec at 72°C; and a final step of 2–10 min at 72°C. DMSO (1.3 – 2.5%) and/or betaine (1 M) was added for some samples. PCR products were purified from a 1% agarose gel, using GFX™ PCR DNA & Gel Band Purification Kit (GE Healthcare), and reamplified if necessary. Gel-extracted PCR products were sequenced directly on a 3730 96-capillary sequencer (Applied Biosystems). Internal primers were used to get complete sequences of both strands.

### Phylogenetic analyses

Sequences were assembled and aligned with the ED editor of the MUST package [58], and manually adjusted taking amino acid properties in consideration. Amino acid repeats and sites not sequenced or gapped in more than 25% of the taxa were excluded from analysis. This resulted in a dataset of 1,101 bp for ADRA2B, 1,161 bp for AR, 852 bp for GHR, and 1,173 bp for vWF. The full data matrix is available from Treebase (accession number: M3679). Phylogenetic reconstructions on each gene separately and on the concatenated dataset were performed by maximum likelihood (ML) with PAUP*, version 4b10 [59], and by Bayesian analyses with MRBAYES, version 3.1.2 [60]. The best fitting model under the ML criterion was selected from the "Akaike Criterion" output of MODELTEST, version 3.7 [61]. The ML analysis was conducted using a loop approach to estimate the best tree and the optimal likelihood parameters. With this approach parameters and best tree are re-estimated until they reach stability. Node stability was estimated by 100 non-parametric bootstrap replicates [62]. A major advantage of Bayesian phylogenetic inference is the possibility of partitioning the data, giving each partition its own best fitting model of sequence evolution. However, overpartitioning may introduce unnecessary sampling variances which could influence the phylogenetic estimates. For the twelve possible codon partitions (each codon position of each gene) MODELTEST was used to calculate the best fitting model of sequence evolution. As further explained in Table 6, codon partitions with similar models and model parameters were merged, resulting in nine partitions for the Bayesian analyses. Two runs of four Markov chains were calculated simultaneously for 1,000,000 generations with initial equal probabilities for all trees and starting with a random tree. Tree sampling frequency was each 20 generations, and the consensus tree with posterior probabilities was calculated after removal of the first 25% of the total number of trees generated, corresponding to 12,500 trees. The average standard deviation of split frequencies between the two independent runs was lower than 0.01.

**Table 6:** Best fitting evolutionary model for each codon position.

			Estimated by MODELTEST								Estimated by PAML		
Gene	CP	Length	π_A_	π_C_	π_G_	Best model	TRatio or Rmat	alpha	PInvar	PN	kappa	alpha	PN

ADRA2B	1	367	0.22	0.31	0.28	K81uf+I+Γ	(1.0 2.5 0.7 0.7 2.5)	1.04	0.36	1	1.05	0.34	1
	2	367	0.19	0.30	0.21	GTR+Γ	(1.6 6.1 0.7 2.6 3.6)	0.24	0	2	1.18	0.20	2
	3	367	0.10	0.42	0.32	TVM+Γ	(1.2 4.4 2.5 0.4 4.4)	2.56	0	3	1.96	1.78	3

AR	1	387	0.22	0.25	0.32	TIM+Γ	(1.0 4.5 0.5 0.5 3.0)	0.59	0	4	1.94	0.54	4
	2	387	0.27	0.31	0.20	TVM+Γ	(1.2 2.9 0.7 1.8 2.9)	0.71	0	5	0.69	0.55	5
	3	387	0.21	0.31	0.23	TIM+Γ	(1.0 5.4 0.7 0.7 4.4)	1.46	0	6	2.34	1.42	3

GHR	1	284	0.26	0.24	0.33	GTR+Γ	(2.1 3.9 0.9 1.1 2.8)	0.71	0	5	0.90	0.59	5
	2	284	0.31	0.31	0.18	HKY+I+Γ	1.74	1.42	0.28	7	1.43	0.52	4
	3	284	0.21	0.32	0.21	TIM+Γ	(1.0 6.0 0.8 0.8 3.8)	2.69	0	6	2.16	2.45	3

vWF	1	391	0.25	0.28	0.32	TVM+Γ	(1.7 3.4 1.1 1.3 3.4)	0.65	0	5	0.89	0.59	5
	2	391	0.29	0.28	0.17	TrN+Γ+I	(1.0 5.6 1.0 1.0 4.3)	0.81	0.31	8	1.97	0.33	1
	3	391	0.09	0.38	0.40	TVM+Γ	(2.5 9.9 5.6 0.8 9.9)	3.14	0	9	3.02	1.92	3

Best models and parameters were found with the akaike criterion as implemented in MODELTEST 3.7 and with PAML, for each codon position of the four gene fragments. Codon positions with similar model and model parameters were regrouped into the same partition, which resulted in nine partitions when estimated by MODELTEST and five partitions when estimated by PAML. Codon positions were merged into the same partition when none of their model parameters (e.g., TRatio of position 1 compared to TRatio of position 2, PInvar 1 to PInvar 2, etc.) differed by more than 100%. For the parameters estimated by PAML we took also into account, to define the partitions, the rate of the various gamma low categories; these parameters are not included in this table. TRatio, transition/transversion ratio; Rmat, rate matrix; π, base frequency; PInvar, proportion of invariable sites; alpha, shape of gamma distribution; kappa, value of the transition/transversion ratio under the F84 model. CP stands for codon position and PN for partition number.

To assess the stability of the phylogenetic position of *Geogale aurita*, our result was compared, according to both Kishino and Hasegawa [63] and Shimodaira and Hasegawa [64] (using RELL bootstrap as well as full optimization methods), to the hypotheses of Olson and Goodman [17] and Asher and Hofreiter [18]. Furthermore, Ka (i.e., number of nonsynonymous substitutions per nonsynonymous site) and Ks (i.e., number of synonymous substitutions per synonymous site) of pairwise tenrec sequences were calculated using the program CODEML from the PAML package [65] in order to assess the molecular divergence between the two *Geogale *GHR sequences and compare it with the level of molecular divergence displayed within the Malagasy tenrec clade.

### Molecular dating

We used the Bayesian approach [66] as implemented in the MULTIDIVTIME program package [52], which relaxes the molecular clock by allowing continuous autocorrelation of substitution rates among the branches of the phylogenetic tree. The concatenated sequence dataset was partitioned into the same nine categories as for the Bayesian phylogenetic analyses, and branch lengths were calculated under the F84 + Γ model of sequence evolution, which is the most complex model available in MULTIDIVTIME. Each of the described analyses was run twice in order to assess the consistency of the results. The prior for the root was set at 100 Mya, however, analyses with 65 Mya, 80 Mya and 120 Mya as prior age were also performed in order to estimate the impact of the root prior on our results. For each node, we calculated the variance of the estimated ages over all the runs. A maximal variance of 2*10^-4 ^was found showing that changing the root prior does not influence age estimates. Markov Chain Monte Carlo analyses were run for 1,000,000 generations after a "burn in" of 100,000 generations. The chains were sampled every 100 generations. To assess the influence of a particular partitioning on the dating results, we performed additional analyses using four partitioning schemes: without partitioning, with nine partitions following the results of MODELTEST, with five partitions following the results of ESTBRANCHES using the F84 + Γ model, and with a maximum number of partitions (i.e., twelve). The results of these analyses were close to each other. Notably, all datings for the nodes of interest remained within the 95% credibility intervals of the datings obtained in the analysis using five partitions.

Six well established fossil constraints on divergence times were used: (i) a minimum of 54 and a maximum of 65 Mya for the base of Paenungulata [67]; (ii) a minimum of 50 and a maximum of 63 Mya for the split between feliform and caniform Carnivora [45,68]; (iii) a minimum of 54 and a maximum of 58 Mya for the split between hippomorph and ceratomorph Perissodactyla [69]; (iv) a minimum of 55 and a maximum of 65 Mya for the base of Cetartiodactyla [70]; (v) a minimum of 37 Mya for the split between ochotonids and leporids [45]; (vi) a minimum of 60.5 and a maximum of 100.5 Mya for the divergence time between rodents and primates [71]. To assess the reciprocal consistency of all calibration points we used the cross-validation method described in [16]. In this method each calibration point is removed in turn and the remaining calibration points are used to estimate its age. Calibration points, for which the estimated and paleontological dates are not congruent, are considered as inconsistent and are consequently removed from the analyses.

## Authors' contributions

MV and WWdJ initiated the study. JG contributed to collect specimens in the field, CP obtained DNA sequences, CP and OM assembled the data, designed and ran the calculations. CP wrote the manuscript and OM, WWdJ and MV helped to improve it. All authors read and approved the final manuscript.

## Supplementary Material

Additional file 1 Calibration points compatibility analysis. Posterior age estimates for all nodes as numbered in additional file 3. The nine partitions were defined as explained in Methods. All calibrations points were used in the first result column whereas in the following ones calibration points were removed in turn from the analyses in order to estimate their impact on node ages. Standard deviations (SD) are given. ^a ^The mean percentage difference represents the average, over all the tree nodes, of the percentage difference between the posterior age estimate calculated with all the calibration points and the one calculated with one calibration point removed. Bold numbers indicates the estimated age of a calibration point when its age constraint was removed from the analyses. Click here for file

Additional file 2 Tree nodes numbering. The figure displays the numbers given to each node in the chronogram. Black circles indicate nodes for which a paleontological time constraint was applied. Click here for file

Additional file 3 Primer sequences. This is the list of the primers used in this study for PCR and sequencing of the GHR and vWF genes. Click here for file
