# Supplementary material for: Molecular phylogeny and divergence times of Malagasy tenrecs: Influence of data partitioning and taxon sampling on dating analyses
Source: BMC Evol Biol. 2008 Mar 31;8:102. doi: 10.1186/1471-2148-8-102 (PMC2330147; doi:10.1186/1471-2148-8-102)
Supplement: Additional file 1 — Calibration points compatibility analysis. Posterior age estimates for all nodes as numbered in additional file 3. The nine partitions were defined as explained in Methods. All calibrations points were used in the first result column whereas in the following ones calibration points were removed in turn from the analyses in order to estimate their impact on node ages. Standard deviations (SD) are given. a The mean percentage difference represents the average, over all the tree nodes, of the percentage difference between the posterior age estimate calculated with all the calibration points and the one calculated with one calibration point removed. Bold numbers indicates the estimated age of a calibration point when its age constraint was removed from the analyses. [file 1471-2148-8-102-S1.doc]

### **Additional file 1 – Calibration points compatibility analysis.**

Posterior age estimates for all nodes as numbered in additional file 3. The nine partitions were defined as explained in Methods. All calibrations points were used in the first result column whereas in the following ones calibration points were removed in turn from the analyses in order to estimate their impact on node ages. Standard deviations (SD) are given. ^a^ The mean percentage difference represents the average, over all the tree nodes, of the percentage difference between the posterior age estimate calculated with all the calibration points and the one calculated with one calibration point removed. Bold numbers indicates the estimated node age of a calibration point when its age constraint was removed from the analyses.

| Nodes | Calibration time frame  (Mya) | Posterior age estimate  (Mya ± s.d.) | Removal of the following calibration point during the analysis | | | | | |
| --- | --- | --- | --- | --- | --- | --- | --- | --- |
|  |  |  | Paenun-gulata | Caniformia/  Feliforma split | Perissodac-tyla | Cetartiodac-tyla | Ochotona/  Leporidae  split | Rodentia/  Primates  split |
| 1 |  | 8.6 ± 1.4 | 9.2 ± 1.7 | 8.6 ± 1.5 | 8. 6 ± 1.5 | 8.5 ± 1.5 | 8.6 ± 1.5 | 8.6 ± 1.5 |
| 2 |  | 10.8 ± 1.7 | 11.6 ± 2.0 | 10.8 ± 1.7 | 10.8 ± 1.7 | 10.8 ± 1.7 | 10.8 ± 1.7 | 10.8 ± 1.7 |
| 3 |  | 22.0 ± 2.5 | 23.6 ± 3.1 | 22.0 ± 2.6 | 22.0 ± 2.6 | 21.9 ± 2.6 | 22.0 ± 2.6 | 22.1 ± 2.6 |
| 4 |  | 23.8 ± 2.6 | 25.6 ± 3.2 | 23.8 ± 2.7 | 23.8 ± 2.7 | 23.7 ± 2.7 | 23.8 ± 2.7 | 23.9 ± 2.7 |
| 5 |  | 6.6 ± 1.2 | 7.1 ± 1.5 | 6.5 ± 1.3 | 6.5 ± 1.3 | 6.5 ± 1.3 | 6.6 ± 1.3 | 6.6 ± 1.3 |
| 6 |  | 16.2 ± 2.1 | 17.5 ± 2.5 | 16.1 ± 2.2 | 16.1 ± 2.2 | 16.1 ± 2.2 | 16.2 ± 2.2 | 16.2 ± 2.2 |
| 7 |  | 20.1 ± 2.4 | 21.7 ± 2.9 | 20.1 ± 2.5 | 20.0 ± 2.4 | 20.1 ± 2.4 | 20.1 ± 2.4 | 20.1 ± 2.4 |
| 8 |  | 29.4 ± 2.9 | 31.6 ± 3.6 | 29.3 ± 3.0 | 29.3 ± 3.0 | 29.3 ± 3.0 | 29.4 ± 3.0 | 29.4 ± 2.9 |
| 9 |  | 47.4 ± 3.7 | 51.0 ± 4.9 | 47.3 ± 3.8 | 47.2 ± 3.9 | 47.2 ± 3.8 | 47.3 ± 3.8 | 47.4 ± 3.8 |
| 10 |  | 69.4 ± 4.0 | 74.4 ± 5.8 | 69.1 ± 4.1 | 68.9 ± 4.1 | 68.9 ± 4.1 | 69.3 ± 4.1 | 69.3 ± 4.0 |
| 11 |  | 72.9 ± 3.9 | 78.2 ± 5.9 | 72.6 ± 4.0 | 72.5 ± 4.1 | 72.5 ± 4.0 | 72.8 ± 4.0 | 72.8 ± 4.0 |
| 12 |  | 76.5 ± 3.6 | 82.0 ± 5.8 | 76.1 ± 3.7 | 76.0 ± 3.8 | 76.0 ± 3.7 | 76.4 ± 3.7 | 76.4 ± 3.6 |
| 13 |  | 58.2 ± 2.9 | 63.7 ± 5.3 | 58.1 ± 3.0 | 58.0 ± 3.0 | 58.0 ± 3.0 | 58.2 ± 3.0 | 58.2 ± 3.0 |
| 14  Paenungulata | 54-65 | 61.9 ± 2.4 | **67. 8 ± 5.3** | 61.7 ± 2.5 | 61.6 ± 2.5 | 61.7 ± 2.5 | 61.8 ± 2.4 | 61.9 ± 2.4 |
| 15 |  | 78.3 ± 3.5 | 84.0 ± 5.8 | 78.0 ± 3.6 | 77.8 ± 3.7 | 77.8 ± 3.6 | 78.2 ± 3.6 | 78.2 ± 3.5 |
| 16 Feliformia/  Caniformia split | 50-63 | 53.3 ± 2.4 | 53.8 ± 2.7 | **50.8 ± 3.8** | 53.0 ± 2.4 | 53.1 ± 2.4 | 53.3 ± 2.5 | 53.3 ± 2.4 |
| 17  Perissodactyla | 54-58 | 55.8 ± 1.1 | 55.9 ± 1.1 | 55.7 ± 1.1 | **53.9 ± 3.9** | 55.7 ± 1.1 | 55.1 ± 1.1 | 55.8 ± 1.1 |
| 18 |  | 80.0 ± 2.8 | 81.4 ± 3.1 | 78.9 ± 3.1 | 78.9 ± 3.5 | 79.1 ± 3.1 | 79.9 ± 2.8 | 79.9 ± 2.9 |
| 19 |  | 53.7 ± 3.0 | 54.2 ± 3.1 | 53.3 ± 3.0 | 53.4 ± 3.0 | 52.1 ± 4.0 | 53.6 ± 3.0 | 53.7 ± 3.0 |
| 20 Cetartiodactyla | 55-65 | 58.5 ± 2.3 | 59.1 ± 2.5 | 58.2 ± 2.3 | 58.2 ± 2.4 | **56.7 ± 3.8** | 58.5 ± 2.4 | 58.5 ± 2.4 |
| 21 |  | 82.4 ± 3.0 | 84.1 ± 3.4 | 81.4 ± 3.2 | 81.4 ± 3.6 | 81.4 ± 3.4 | 82.3 ± 3.1 | 82.3 ± 3.1 |
| 22 |  | 88.1 ± 3.7 | 90.3 ± 4.0 | 87.1 ± 3.8 | 87.1 ± 4.1 | 87.1 ± 3.9 | 87.9 ± 3.7 | 88.0 ± 3.7 |
| 23 |  | 15.9 ± 2.6 | 16.4 ± 2.7 | 15.7 ± 2.6 | 15.8 ± 2.6 | 15.7 ± 2.6 | 15.9 ± 2.6 | 16.0 ± 2.6 |
| 24 Ochotona/  Leporidae split | > 37 | 52.5 ± 4.3 | 54.1 ± 4.5 | 52.0 ± 4.4 | 52.1 ± 4.5 | 52.0 ± 4.4 | **52.4 ± 4.4** | 52.5 ± 4.4 |
| 25 |  | 73.9 ± 4.7 | 76.3 ± 4.9 | 73.2 ± 4.8 | 73.4 ± 4.9 | 73.3 ± 4.8 | 73.8 ± 4.6 | 74.0 ± 4.8 |
| 26 |  | 78.6 ± 4.5 | 81.0 ± 4.7 | 77.8 ± 4.6 | 78.0 ± 4.7 | 77.9 ± 4.6 | 78.5 ± 4.5 | 78.6 ± 4.6 |
| 27 |  | 86.7 ± 4.3 | 89.4 ± 4.6 | 85.8 ± 4.5 | 86.0 ± 4.6 | 85.9 ± 4.5 | 86.6 ± 4.3 | 86.7 ± 4.4 |
| 28 |  | 75.5 ± 4.3 | 77.9 ± 4.6 | 74.8 ± 4.4 | 74.9 ± 4.6 | 74.8 ± 4.5 | 75.5 ± 4.3 | 75.6 ± 4.4 |
| 29 |  | 83.2 ± 4.4 | 85.7 ± 4.7 | 82.4 ± 4.5 | 82.3 ± 4.7 | 82.3 ± 4.6 | 83.1 ± 4.4 | 83.1 ± 4.5 |
| 30 |  | 85.7 ± 4.2 | 88.4 ± 4.5 | 84.9 ± 4.3 | 84.9 ± 4.5 | 84.9 ± 4.4 | 85.7 ± 4.2 | 85.7 ± 4.3 |
| 31 Rodentia/  Primates split | 61.5-100.5 | 89.3 ± 4.2 | 92.1 ± 4.4 | 88.5 ± 4.3 | 88.5 ± 4.5 | 88.5 ± 4.4 | 89.2 ± 4.1 | **89.3 ± 4.3** |
| 32 |  | 96.2 ± 4.1 | 99.2 ± 4.6 | 95.3 ± 4.3 | 95.3 ± 4.5 | 95.2 ± 4.4 | 96.1 ± 4.1 | 96.1 ± 4.2 |
| 33 |  | 99.6 ± 4.4 | 103.2 ± 5.1 | 98.7 ± 4.5 | 98.6 ± 4.7 | 98.6 ± 4.6 | 99.4 ± 4.3 | 99.5 ± 4.5 |
| 34 (root age) |  | 104.1 ± 4.6 | 109.1 ± 6.0 | 103.3 ± 4.7 | 103.2 ± 4.8 | 103.2 ± 4.8 | 103.9 ± 4.5 | 103.9 ± 4.6 |
| Mean percentage difference ^a^ |  |  | 4.8 ± 2.8 | 0.8 ± 0.8 | 0.8 ± 0.6 | 0.9 ± 0.6 | 0.1 ± 0.1 | 0.1 ± 0.1 |
