# Supplementary material for: Molecular phylogeny and divergence times of Malagasy tenrecs: Influence of data partitioning and taxon sampling on dating analyses
Source: BMC Evol Biol. 2008 Mar 31;8:102. doi: 10.1186/1471-2148-8-102 (PMC2330147; doi:10.1186/1471-2148-8-102)
Supplement: Additional file 2 — Tree nodes numbering. The figure displays the numbers given to each node in the chronogram. Black circles indicate nodes for which a paleontological time constraint was applied. [file 1471-2148-8-102-S2.doc]

### **Additional file 2 – Tree nodes numbering.**

Xenarthra

Tupaia

*Cynocephalus variegatus*

*Homo sapiens*

Eulemur

*Cavia porcellus*

Sciurus

*Mus musculus*

*Ochotona princeps*

*Oryctolagus cuniculus*

Lepus

Eulipotyphla

Chiroptera

Lama

*Sus scrofa*

*Physeter catodon*

Equus

Rhinocerotidae

*Cryptoprocta ferox*

*Canis familiaris*

*Elephas maximus*

Trichechus

*Procavia capensis*

*Orycteropus afer*

*Macroscelides proboscideus*

Chrysochloridae

*Micropotamogale lamottei*

*Tenrec ecaudatus*

*Hemicentetes semispinosus*

*Setifer setosus*

*Echinops telfairi*

*Geogale aurita*

*Oryzorictes hova*

*Microgale brevicaudata*

*Microgale pusilla*

*Limnogale mergulus*

1

3

2

4

6

7

8

9

5

10

11

12

13

14

15

16

17

18

19

20

21

22

23

24

25

26

27

28

29

30

31

32

33
