# Supplementary material for: Molecular phylogeny and divergence times of Malagasy tenrecs: Influence of data partitioning and taxon sampling on dating analyses
Source: BMC Evol Biol. 2008 Mar 31;8:102. doi: 10.1186/1471-2148-8-102 (PMC2330147; doi:10.1186/1471-2148-8-102)
Supplement: Additional file 3 — Primer sequences. This is the list of the primers used in this study for PCR and sequencing of the GHR and vWF genes. [file 1471-2148-8-102-S3.doc]

### **Additional file 3 – Primer sequences**

GHR PCR and sequencing primers:

GHR-For: 5’-TGG GTT GAR TTY ATY GAR CTR GAT ATT G-3’

GHR-Rev: 5’-GCA TGA TTT TGT TCA GTT GGT CTG TGC-3’

GHR-F2: 5’-GTG TGA CAT GCM TCC TGA AGT G-3’

GHR-R2: 5’-CAC TTC AGG AKG CAT GTC ACA C-3’

vWF PCR primers:

vWF-ForMicro: 5’-GCA AGC TGC TCG ACC TGG TCT TC-3’

vWF-RevMicro: 5’-GCA GCC GCT TGA TCT CRT CAG AGG-3’
